# EphrinB1 promotes cancer cell migration and invasion through the interaction with RhoGDI1

**DOI:** 10.1038/onc.2017.386

**Published:** 2017-10-23

**Authors:** H J Cho, Y-S Hwang, J Yoon, M Lee, H G Lee, I O Daar

**Affiliations:** 1 Immunotherapy Convergence Research Center, Korea Research Institute of Bioscience and Biotechnology, Yuseong-gu, Daejeon, Korea; 2 Cancer & Developmental Biology Laboratory, National Cancer Institute, National Institutes of Health, Frederick, MD, USA

## Abstract

Eph receptors and their corresponding ephrin ligands have been associated with regulating cell–cell adhesion and motility, and thus have a critical role in various biological processes including tissue morphogenesis and homeostasis, as well as pathogenesis of several diseases. Aberrant regulation of Eph/ephrin signaling pathways is implicated in tumor progression of various human cancers. Here, we show that a Rho family GTPase regulator, Rho guanine nucleotide dissociation inhibitor 1 (RhoGDI1), can interact with ephrinB1, and this interaction is enhanced upon binding the extracellular domain of the cognate EphB2 receptor. Deletion mutagenesis revealed that amino acids 327–334 of the ephrinB1 intracellular domain are critical for the interaction with RhoGDI1. Stimulation with an EphB2 extracellular domain-Fc fusion protein (EphB2-Fc) induces RhoA activation and enhances the motility as well as invasiveness of wild-type ephrinB1-expressing cells. These Eph-Fc-induced effects were markedly diminished in cells expressing the mutant ephrinB1 construct (Δ327–334) that is ineffective at interacting with RhoGDI1. Furthermore, ephrinB1 depletion by siRNA suppresses EphB2-Fc-induced RhoA activation, and reduces motility and invasiveness of the SW480 and Hs578T human cancer cell lines. Our study connects the interaction between RhoGDI1 and ephrinB1 to the promotion of cancer cell behavior associated with tumor progression. This interaction may represent a therapeutic target in cancers that express ephrinB1.

## Introduction

The Eph receptors and their cognate ligands, ephrins, are implicated in the regulation of a number of biological processes such as axon guidance, formation of tissue-boundary, angiogenesis and cell migration.^1, 2, 3^ Ephrins are separated into two subclasses; one subclass represented by five A-type ligands that are glycosylphosphatidylinositol (GPI)-linked to the membrane, and another represented by three B-type transmembrane proteins with relatively short cytoplasmic domains. There are nine A-type and five B-type Eph receptors that are distinguished on the basis of their sequence, and affinity for the A and B ligands.^4^ The interactions between Eph receptors residing on one cell and ephrins on another cell results in bi-directional signaling where the activated receptor tyrosine kinase transduces intracellular signals (‘forward’ signaling), while the ligand acts as a scaffold to transmit signals in its host cell (‘reverse’ signaling).^3^ The de-regulation of the Eph/ephrin signaling pathway has been implicated in tumorigenesis and metastasis in a number of human cancers, such as breast, gastric, prostate, lung and colon cancer.^5, 6, 7, 8^ Generally, it appears that increased Eph forward signaling is commonly tumor suppressive, whereas ephrin reverse signaling is often correlated with the promotion of malignancy.^7^

The transmembrane ephrinB proteins ‘reverse’ signal through a scaffolding function imparted through the intracellular domain that recruits various signaling proteins.^3^ There are several signaling molecules reported to be associated with ephrinBs in a phosphorylation-independent manner and transmit reverse signaling of ephrinB1. Among these are the GTP exchange factor PDZ-RGS3, the gap junction communication protein CX43, a major Wnt signaling platform (dishevelled), a key scaffold protein in the Par polarity complex known as the partitioning defective protein 6 (Par-6), as well as a zinc finger protein (ZXH2).^9, 10, 11, 12, 13, 14^ Phosphorylation-dependent interactions between ephrinBs and the signaling mediators Grb4 and STAT3 have been identified that promote functional effects on cell morphology.^15, 16^ One group of key components of ephrinB reverse signaling is the RhoGTPases, which have been shown to control cytoskeletal dynamics, cell polarity and cell movement.^17^ Although we have previously shown that Connector Enhancer of Kinase Suppressor of Ras1 (CNK1) is an ephrinB1-associated protein that bridges ephrinB1 with RhoA-dependent JNK activation and cell migration,^18^ the mechanism by which ephrinB1 regulates RhoA remains to be revealed.

The RhoGTPase family of small G proteins transduce extracellular signals to downstream effectors. These proteins control diverse cell processes including proliferation, cytoskeletal rearrangements, cell adhesion, cell motility, axon guidance and vesicle trafficking.^19, 20^ Anomalous signaling by RhoGTPases is implicated in severe cellular disorders, such as neurological abnormalities, immunological diseases and malignant transformation.^21^ RhoGTPases cycle between a cytoplasmic inactive guanosine diphosphate (GDP)-bound form and a membrane localized active guanosine triphosphate (GTP)-bound form. This cycle is tightly regulated by several proteins, that include guanine nucleotide exchange factors (GEFs) that stimulate the exchange of GDP for GTP, thereby activating RhoGTPases; GTPase-activating proteins (GAPs) that catalyze GTP hydrolysis, resulting in the inactivation of RhoGTPases; and Rho guanine nucleotide dissociation inhibitors (RhoGDIs) that bind to RhoGTPases and regulate their spatiotemporal activity.^22, 23^ The RhoGDI family consists of three members in mammals (RhoGDI1, 2 and 3). RhoGDI1 is ubiquitously expressed,^24^ while RhoGDI2 is found predominantly in hematopoietic cells.^25, 26^ RhoGDI3 is expressed in the brain, lung, kidney and pancreas.^27, 28^ In the cytoplasm, RhoGDIs bind to most RhoGTPases, which maintains these proteins in the inactive form. As a result of this binding, RhoGDIs prevent RhoGTPases from interacting with GEFs or their effector proteins. When RhoGTPases are disengaged from RhoGDIs, they are able to integrate into the plasma membrane and are activated by membrane-associated GEFs.^29, 30^ The interaction of RhoGTPases with RhoGDIs is regulated by several mechanisms: phosphorylation, phospholipids and protein–protein interactions. Phosphorylation of RhoGDIs by protein kinases, such as PKCα, PAK1 and Src, decreases their affinity for RhoGTPases, promoting the release of RhoGTPases and allowing subsequent activation by a RhoGEF.^31, 32, 33, 34^ Specific phospholipids, such as phosphatidic acid and anionic phospholipids, promote dissociation of the RhoGTPases from RhoGDIs.^35, 36^ Specific protein interactions also allow the displacement of RhoGTPases from complexes with RhoGDIs. For example, ezrin/radixin/moesin (ERM) family proteins,^37^ the tyrosine kinase Etk,^38^ p75 neurotrophin receptor^39^ and TROY (An orphan receptor of the tumor necrosis factor family)^40^ allow the dissociation and subsequent activation of RhoGTPases.

In the current study, we provide mechanistic insight into the regulation of Rho activity by ephrinB1. We identified RhoGDI1 as an ephrinB1-associated protein that has enhanced interactions with ephrinB1 upon stimulation with EphB2-Fc. Moreover, this heightened association between ephrinB1 and RhoGDI1 promotes cell migration and invasion through RhoA activation.

## Results

### EphrinB1 is associated with RhoGDI1

**Figure 1:**
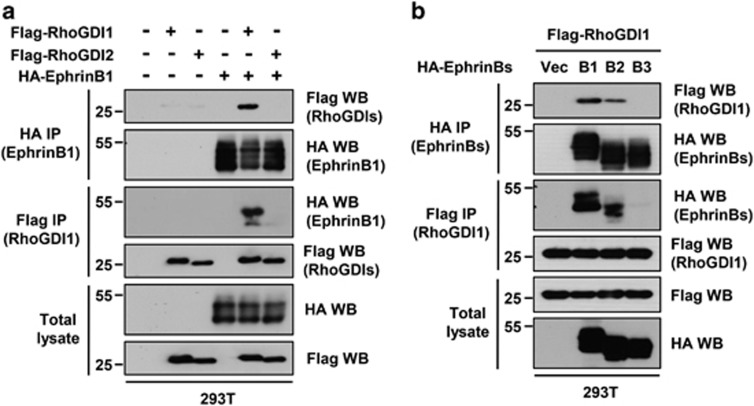
EphrinB1 and ephrinB2 interacts with RhoGDI1 but not RhoGDI2. (**a**) HEK293T cells were co-transfected HA-tagged ephrinB1 with Flag-tagged RhoGDI2 or RhoGDI2 as indicated. Cell lysates were immunoprecipitated with HA or Flag antibodies. Immunoprecipitates and total lysates were immunoblotted with HA or Flag antibodies. (**b**) Flag-tagged RhoGDI1 was co-transfected into HEK293T cells with HA-tagged ephrinB1, ephrinB2 or ephrinB3 as indicated. Cell lysates were immunoprecipitated with HA or Flag antibodies. Immunoprecipitates and total lysates were immunoblotted with HA or Flag antibodies.

The dissociation of RhoGTPases from RhoGDIs is a prerequisite for their activation and the interaction of RhoGDIs with specific receptor proteins, such as p75 neurotrophin receptor and TROY, regulate this dissociation.^39, 40^ Since the activity of RhoGTPases is critical for ephrinB1-mediated reverse signaling,^13, 18^ we speculated that ephrinB1 may associate with RhoGDIs. To investigate this possibility, HEK293T cells were transfected with HA-tagged ephrinB1 and Flag-tagged RhoGDI1 or RhoGDI2 constructs, and cell lysates were immunoprecipitated with HA or Flag antibodies. Flag-tagged RhoGDI1 was found in the HA-tagged ephrinB1 immune-complexes, and HA-tagged ephrinB1 was located in Flag-tagged RhoGDI1 immune-complexes (Figure 1a). However, RhoGDI2 was not found in ephrinB1 immunoprecipitates and vice versa (Figure 1a), suggesting that ephrinB1 interacts with RhoGDI1 but not RhoGDI2. Next, we assessed whether RhoGDI1 associates specifically with ephrinB1. We performed reciprocal immunoprecipitation using cell extracts from HEK293T cells containing Flag-tagged RhoGDI1 and HA-tagged ephrinB1, ephrinB2 or ephrinB3. The data indicated that RhoGDI1 binds robustly to ephrinB2, and less well with ephrinB1, whereas ephrinB3 did not interact with RhoGDI1 at all (Figure 1b).

**Figure 2:**
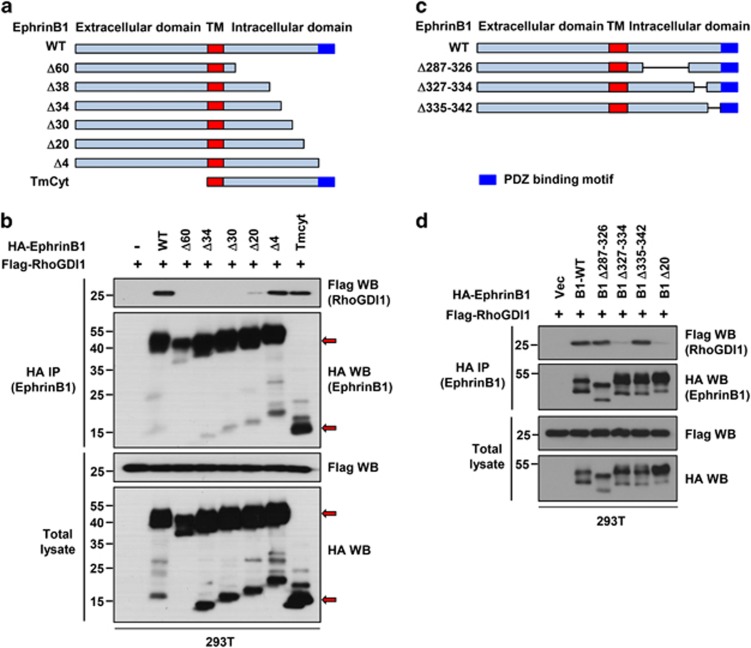
Mapping the region of ephrinB1 for RhoGDI1 interaction by deletion mutagenesis. (**a**) Schematic representation of wt ephrinB1 or deletion mutants lacking 4, 20, 30, 34 or 60 amino acids from the C terminus or the extracellular domain (TmCyt). (**b**) HA-tagged wt or deletion mutants of ephrinB1 were co-transfected with Flag-tagged RhoGDI1 into HEK293T cells as indicated. Cell lysates were immunoprecipitated with HA antibody. Immunoprecipitates and total lysates were immunoblotted with HA or Flag antibodies. (**c**) Schematic representation of wt ephrinB1 or deletion mutants lacking amino acid 287–326, 327–334 or 335–342 of ephrinB1 C terminus. (**d**) HEK293T cells were co-transfected Flag-tagged RhoGDI1 along with wt or deletion mutants of ephrinB1 as indicated. Cell lysates were immunoprecipitated with HA antibody. Immunoprecipitates and total lysates were immunoblotted with HA or Flag antibodies.

To verify the region of ephrinB1 responsible for the association with RhoGDI1, we constructed a series of C-terminal truncation mutants of ephrinB1 (Figure 2a). HEK293T cells were co-transfected with deletion mutants of ephrinB1 along with RhoGDI1, and cell lysates were subjected to coimmunoprecipitation (Co-IP) followed by western blot analysis. Unlike wild-type eprhinB1 (wt), deletion of 20 amino acids from the C terminus of ephrinB1 significantly inhibited the association between the two proteins. Interestingly, a deletion mutant of the C-terminal four amino acids (Δ4-ephrinB1) that removes the PDZ-binding motif, as well as a mutant consisting of the transmembrane and cytoplasmic domain (Tm-cyt) displayed similar interactions as the wild-type ephrinB1 with RhoGDI1 (Figure 2b). To more clearly define which amino acids within the C-terminal 20 amino acids were necessary for an interaction with RhoGDI1, we created additional internal deletion mutants in ephrinB1 that also retain the PDZ-binding motif (Figure 2c). These mutants of ephrinB1 were individually co-tranfected along with RhoGDI1 into HEK293T cells. Lysates were prepared and Co-IP analysis was performed. Deletion of amino acids 327–334 of ephrinB1 disrupted the association between ephrinB1 with RhoGDI1, but removing amino acids 287–326 or 335–342 of ephrinB1 had no substantive effect (Figure 2d). These data indicate that the region consisting of amino acids 327–334 of ephrinB1 is indispensable for the interaction with RhoGDI1.

### EphrinB1/RhoGDI1 interaction is enhanced by EphB2-Fc

**Figure 3:**
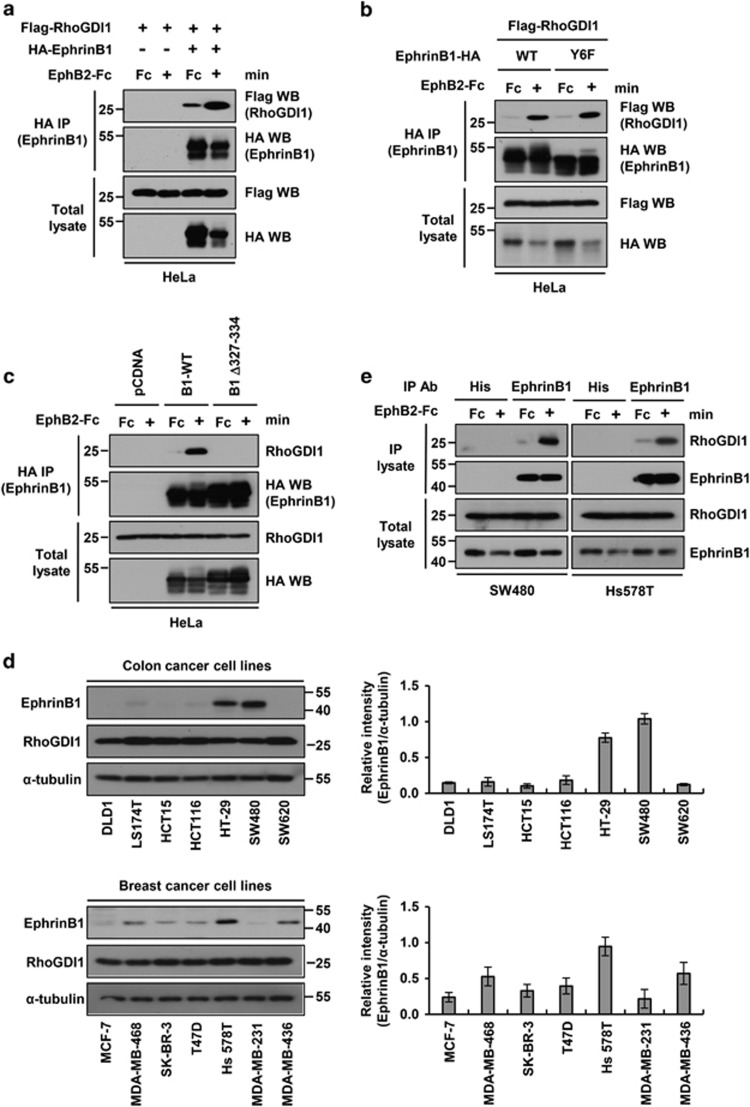
EphB2-Fc stimulation enhances the interaction between ephrinB1 and RhoGDI1. (**a**) HeLa cells were co-transfected Flag-RhoGDI1 with HA-tagged ephrinB. 30 hrs after transfection, cells were serum-starved for 18 h and then were treated with 2.5 μg/ml of control Fc or EphB2 ectodomain-Fc fusion for 30 min. Lysates were subjected to IP and immunoblot analysis with indicated antibodies. (**b**) HeLa cells were transfected with wt or Y6F mutant ephrinB1 and were treated with 2.5 μg/ml of control Fc or EphB2-Fc for 30 min. lysates were subjected to IP and immunoblot analysis with indicated antibodies. (**c**) HeLa cells were transfected with wt or 327–334 deletion mutant of ephrinB1 and were treated with control Fc or EphB2-Fc for 30 min. lysates were subjected to IP and immunoblot analysis with indicated antibodies. (**d**) Representative western blots of ephrinB1 and RhoGDI1 in human colon and breast cancer cell lines (left panels). Relative intensities were measured by ImageJ and calculated using ephrinB1;α-tubulin ratios (histograms). Data represent the mean±s.d. of three individual experiments. (**e**) SW480 and Hs578T cells were treated with 2.5 μg/ml of control Fc or EphB2-Fc for 30 min. Cell lysates were immunoprecipitated with His (as control) or ephrinB1 antibodies. Immunoprecipitates and total lysates were immunoblotted with indicated antibodies.

Since ephrinB1 transduces a reverse signal upon engagement with its corresponding Eph receptors,^16^ we next verified whether the stimulation by an EphB receptor affects the interaction of ephrinB1 with RhoGDI1. HeLa cells, which have nearly undetectable levels of endogenous ephrinB1,^18^ were co-transfected with ephrinB1 and RhoGDI1 and then treated with the Fc portion of human Ig (Fc) or EphB2 ectodomain fused to human Fc (EphB2-Fc). Co-IP analysis was performed on the cell lysates. The treatment with EphB2-Fc but not control Fc significantly enhanced the ephrinB1/RhoGDI1 interaction (Figure 3a). Because ephrinB1 can be phosphorylated at six possible conserved tyrosine residues within the intracellular domain following EphB2 engagement,^2, 3^ we next employed an ephrinB1 mutant with the six conserved tyrosines changed to phenylalanine (Y6F), to effectively inhibit ephrinB1 tyrosine phosphorylation. This construct allowed us to test whether tyrosine phosphorylation of ephrinB1 was responsible for enhancing the association with RhoGDI1. HeLa cells were transfected with wild-type ephrinB1 or the Y6F mutant. After 30 min of treatment with an Fc control or EphB2-Fc, cells were lysed and subjected to immunoprecipitation and western analysis. Upon stimulation with the EphB2-Fc, an increase in RhoGDI1 was detected in the Y6F ephrinB1 mutant immune-complexes as well as wild-type ephrinB1 immune-complexes (Figure 3b), suggesting that the EphB2-Fc-induced interaction between ephrinB1 and RhoGDI1 is independent of tyrosine phosphorylation. Having established that amino acids 327–334 of ephrinB1 were critical for an interaction between exogenously expressed ephrinB1 and RhoGDI1, and that binding of the cognate EphB2 receptor enhances the interaction, we examined whether the endogenous RhoGDI1 displayed similar properties. HeLa cells stably expressing a vector control or wt ephrinB1 or a Δ327–334 mutant of ephrinB1 were subjected to immunoprecipitation with HA antibody. The treatment with EphB2-Fc significantly enhances the interaction of endogenous RhoGDI1 with wt ephrinB1 but not the Δ327–334 mutant of ephrinB1 (Figure 3c), indicating that the region encompassing amino acids 327–334 of ephrinB1 are required for an interaction with RhoGDI1 induced by EphB2-Fc. Next, we surveyed the expression of ephrinB1 and RhoGDI1 in colon and breast cancer cell lines. Western analysis showed that ephrinB1 is expressed in 2 out of 7 human colorectal cancer cell lines (SW480 and HT-29 cells) and 5 out of 7 breast cancer cell lines, while RhoGDI1 is detected in all cancer cell lines (Figure 3d). To verify the association between ephrinB1 and RhoGDI1 can occur with endogenous proteins, we used the SW480 colon cancer and Hs578T breast cancer cell lines that amply express both ephrinB1 and RhoGDI1. The results show that RhoGDI1 was detected in the ephrinB1 immune-complexes from these cell lines treated with EphB2-Fc, indicating that both proteins may partner *in vivo* upon stimulation with EphB2-Fc (Figure 3e).

### EphrinB1 stimulation by EphB2-Fc promotes RhoA activation and the dissociation of RhoGDI1 from RhoA

**Figure 4:**
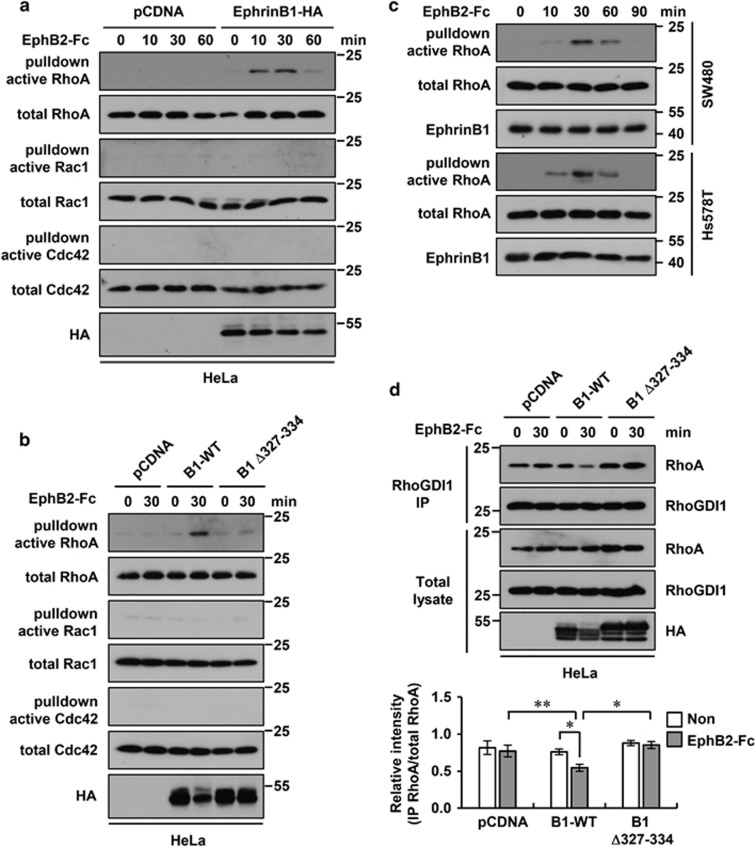
EphrinB1 stimulation by EphB2-Fc promotes RhoA activation and the dissociation RhoGDI1 from RhoA. (**a**) Hela cells were transfected with vector control or HA-tagged ephrinB1 and treated with EphB2-Fc for indicated times. (**b**) HeLa cells were transfected with vector control, wt ephrinB1, or 327–334 deletion mutant of ephrinB1 and treated with EphB2-Fc for 30 min. (**c**) SW480 cells were treated with EphB2-Fc for indicated times. (**a**–**c**) A pull-down assay using Rhotekin-agarose (RhoA) or PAK1-agarose (Rac1/Cdc42) were performed as described in Materials and Methods. Samples and total lysates were immunoblotted with indicated antibodies. (**d**) HeLa cells were transfected with vector control, wt ephrinB1, or 327–334 deletion mutant of ephrinB1 and treated with EphB2-Fc for 30 min. lysates were immunoprecipitated with RhoGDI1 antibody. Immunoprecipitates and total lysates were immunoblotted with indicated antibodies (upper panel). Relative intensities were measured by ImageJ and calculated using immunoprecipitated RhoA;total RhoA ratios (histogram). Data represent the mean±s.d. of three individual experiments. *P<0.05, **P<0.01.

RhoGDI1 is a key regulator of the RhoGTPase family of proteins.^23^ Since stimulation of ephrinB1 by EphB2-Fc promotes ephrinB1/RhoGDI1 association, we assessed whether the activity levels of RhoA, Rac1 and Cdc42 were affected in cells. HeLa cells expressing an empty vector or wt ephrinB1 were treated with EphB2-Fc over a 60 min period and lysates were prepared. Active RhoA and Rac1/Cdc42 were assessed via pull-down assays with either the Rhotekin-binding protein or the Pak1-binding protein, respectively. In the case of wt ephrinB1-expressing HeLa cells, active RhoA levels are increased by EphB2-Fc within 10 min, but EphB2-Fc does not affect active RhoA levels in control cells. Interestingly, active Rac1 and Cdc42 levels are unchanged by EphB2-Fc, irrespective of ephrinB1 expression (Figure 4a). To test whether the activation of RhoA by EphB2-Fc is dependent on the interaction of ephrinB1 with RhoGDI1, we used the Δ327–334 mutant of ephrinB1 that does not interact with RhoGDI1 (Figure 3c). As expected, wt ephrinB1-expressing HeLa cells showed increased RhoA activity upon EphB2-Fc stimulation. Unlike the case of wt ephrinB1, EphB2-Fc treatment of HeLa cells expressing the Δ327–334 mutant of ephrinB1 has no significant effect on the levels of active RhoA, Rac1 and Cdc42 (Figure 4b). To further test the EphB2-Fc induced effect on RhoA activation, we used SW480 and Hs578T cells that endogenously express both ephrinB1 and RhoGDI1 (Figure 3d). Active RhoA reached its peak at 30 min and decreased to basal levels within 90 min after EphB2-Fc treatment in these two cell lines (Figure 4c). We next examined whether the EphB2-Fc-induced interaction of ephrinB1 with RhoGDI1 affects the dissociation of RhoGDI1 from RhoA. Cells were treated with EphB2-Fc for 30 min and lysates were subjected to immunoprecipitation using RhoGDI1 antibody and Western analysis to detect RhoA. The interaction of RhoA with RhoGDI1 is reduced by EphB2-Fc in wt ephrinB1-expressing cells, while it is not altered significantly in the ephrinB1 Δ327–334 mutant-expressing cells as well as control cells (Figure 4d). These findings provide evidence that EphB2-Fc induced activation of RhoA is dependent on the interaction of RhoGDI1 with ephrinB1.

### EphrinB1 stimulation with EphB2-Fc promotes cell migration and invasion through RhoA

**Figure 5:**
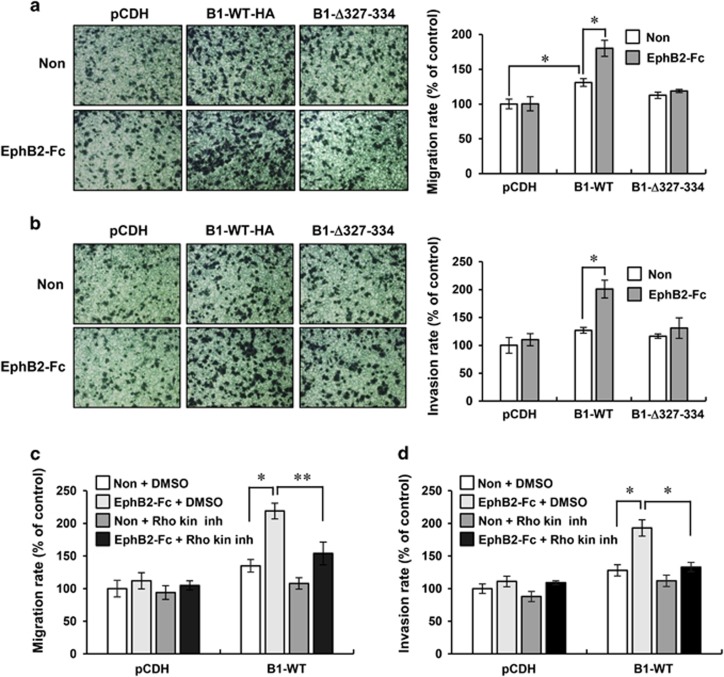
The stimulation of ephrinB1 with EphB2-Fc promotes cell migration and invasion through RhoA. HeLa-derived cell lines stably transfected with an empty (pCDH), wt ephrinB1 (B1-wt), or Δ327–334 mutant of ephrinB1 (B1-Δ327–334)-expressing vector were serum-starved for 16 h. Cells were placed in the inner chamber of transwell and treated with 2.5 μg/ml of Fc control (Non) or EphB2-Fc. Migration assay (**a**) and invasion assay (**b**) were performed as described in Materials and Methods. Representative images of migrating cells stained with crystal violet are displayed (left). Quantitative data of migration and invasion assay are expressed relative to the migration and invasion ability of pCDH cells treated Fc control (right). (**c**, **d**) Serum-starved pCDH or B1-wt cells were placed in the inner chamber of transwell and treated with DMSO (vehicle control) or 10 μm Rho kinase inhibitor in the absence or presence of 2.5 μg/ml of EphB2-Fc as indicated. Migration assay (**c**) and invasion assay (**d**) were performed as described in Materials and Methods. Quantitative data of migration and invasion assay are expressed relative to the migration and invasion ability of pCDH cells treated Fc control in the absence of EphB2-Fc. Data represent the mean±s.d. of three individual experiments. *P<0.05, **P<0.01.

RhoA activity is important for cancer cell migration.^41^ Because we established that EphB2-Fc promoted the activation of RhoA through an interaction between RhoGDI1 and ephrinB1, we assessed whether EphB2-Fc also affected cell motility and invasiveness. A transwell migration assay was performed on HeLa cells that were stably transfected with empty vector, wt ephrinB1, or the Δ327–334 mutant of ephrinB1 in the presence or absence of EphB2-Fc. Although HeLa cells expressing wt ephrinB1 showed slightly increased migration (about 30%) even in the absence of EphB2-Fc, the treatment with EphB2-Fc significantly increased migration (about 80%), compared with the vector control cells (Figure 5a). However, the enhanced migration by EphB2-Fc was considerably reduced in HeLa cells expressing the mutant ephrinB1 (Figure 5a). We next performed a Matrigel invasion assay in these cell lines to investigate whether EphB2-Fc led to enhanced invasion activity. Indeed, wt ephrinB1-expressing cells, but not control vector or mutant ephrinB1-expressing cells, showed enhanced cell invasion upon treatment with EphB2-Fc (Figure 5b). To test whether RhoA activation was necessary for the motility and the invasiveness of these cells, the HeLa cells expressing the vector control or wt ephrinB1 were subjected to transwell migration or invasion assays in the absence or presence of a Rho kinase inhibitor. As expected, the stimulation of ephrinB1 with EphB2-Fc increased migration and invasion in HeLa cells expressing wt ephrinB1. However, this enhanced migration and invasion was reversed by treatment with the Rho kinase inhibitor (Figures 5c and d). Taken together, these data show that ephrinB1 reverse signaling elicited by EphB2-Fc promotes cell motility and invasiveness through the activation of RhoA and these functions may be dependent on the ephrinB1/RhoGDI1 interaction.

### G173V RhoGDI1 mutant abrogates cell migration/invasion and RhoA activation induced by EphB2-Fc

**Figure 6:**
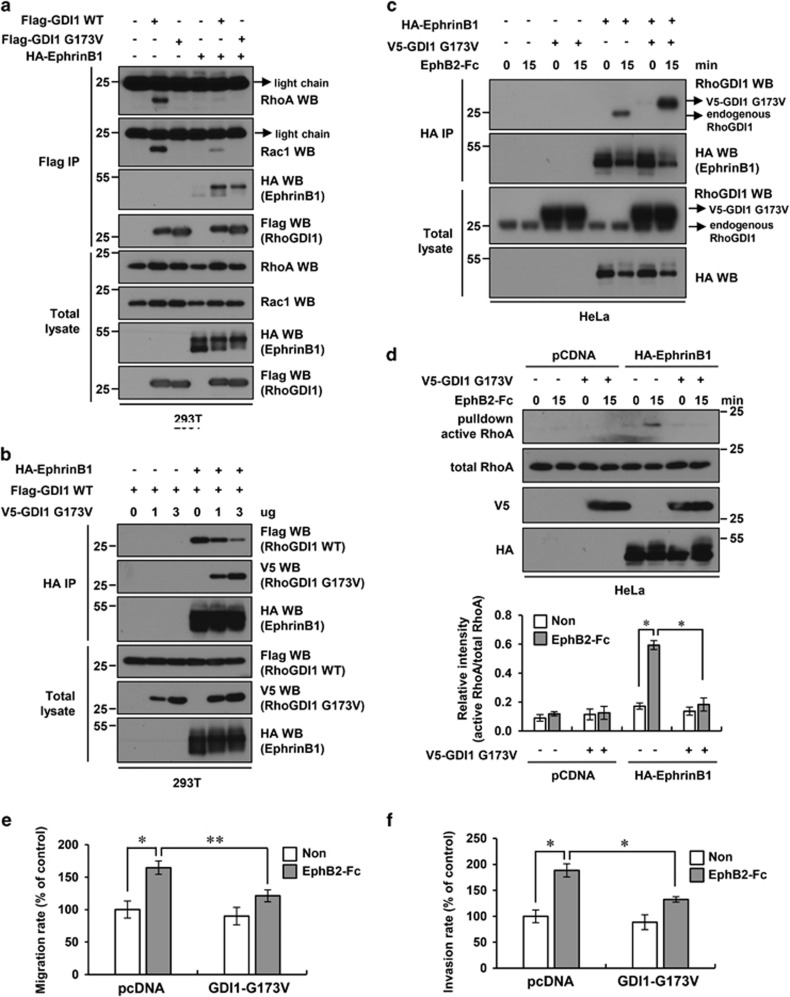
G173V RhoGDI1 mutant abrogates cell migration/invasion and RhoA activation induced by EphB2-Fc. (**a**) HEK293T cells were co-transfected Flag-RhoGDI1 wt, Flag-RhoGDI1 G173V or HA-ephrinB1. Lysates were subjected to IP and immunoblot analysis with indicated antibodies. (**b**) HEK293T cells were co-transfected HA-ephrinB1, Flag-RhoGDI1 wt or V5-RhoGDI1 G173V. Lysates were subjected to IP and immunoblot analysis with indicated antibodies. (**c**) HeLa cells were co-transfected HA-ephrinB1, and/or V5-RhoGDI1 G173V, and treated with 2.5 μg/ml of control Fc or EphB2-Fc for 15 min. lysates were subjected to IP and immunoblot analysis with indicated antibodies. (**d**) HeLa cells were co-transfected HA-ephrinB1 or V5-RhoGDI1 G173V, and treated with 2.5 μg/ml of control Fc or EphB2-Fc for 15 min. A pull-down assay using Rhotekin-agarose (RhoA) were performed as described in Materials and Methods. Samples and total lysates were immunoblotted with indicated antibodies (upper panel). Relative intensities were measured by ImageJ and calculated using active RhoA: total RhoA ratios (histogram). Data represent the mean±s.d. of three individual experiments. *P<0.05. (**e**, **f**) HeLa cells stably expressing wt ephrinB1 were transfected with empty vector or V5-RhoGDI1 G173V. 36 h after transfection, cells were serum-starved overnight and then subjected to migration (**e**) and invasion (**f**) assays as described in Materials and Methods. Data represent the mean±s.d. of three individual experiments. *P<0.05, **P<0.01.

It was recently reported that a G173V mutant RhoGDI1 was identified in nephrotic syndrome patients, and this mutant does not interact with RhoGTPase family proteins, such as RhoA, Rac1 and Cdc42.^42^ If this mutant is still capable of associating with ephrinB1, its overexpression may abrogate the EphB2-Fc-induced interaction of ephrinB1 with endogenous RhoGDI1. To test this hypothesis, we substituted valine for glycine at amino acid 173 in RhoGDI1 (G173V RhoGDI1). Immunoprecipitation analysis was performed on HEK293T cells that were co-transfected with HA-ephrinB1 and wt Flag-RhoGDI1 or the G173 mutant Flag-RhoGDI1. As expected, RhoA and Rac1 were detected in wt RhoGDI1 immune-complexes but not in those of G173V RhoGDI1. However, ephrinB1 was precipitated with G173V RhoGDI1 as well as wt RhoGDI1 (Figure 6a), suggesting that ephrinB1 can associate with both wt RhoGDI1 and G173V RhoGDI1. To test whether these two RhoGDI1s compete for the interaction with ephrinB1, we co-transfected increasing amounts of the G173V mutant V5-RhoGDI1 along with a constant amount of HA-ephrinB1 and wt Flag-RhoGDI1, and performed HA IPs to determine the presence of wt or G173V RhoGDI1 in the ephrinB1 immune-complexes. As the expressed amounts of G173V RhoGDI1 increase, the abundance of G173V RhoGDI1 in the ephrinB1 immune-complexes also increases, but the amounts of associated wt RhoGDI1 diminish (Figure 6b). As a further test of the competitive inhibition of ephrinB1 signaling by G173V RhoGDI1, we expressed ephrinB1 alone or along with the G173V RhoGDI1 mutant in HeLa cells. After treatment with EphB2-Fc for 15 min, cell lysates were generated and subjected to immunoprecipitation analysis. Western blots of these complexes showed that the interaction of endogenous RhoGDI1 with ephrinB1 is significantly increased by EphB2-Fc treatment in the absence of G173V RhoGDI1. However, the enhanced endogenous RhoGDI1/ephrinB1 interaction elicited by stimulation with EphB2-Fc was markedly reduced when G173V RhoGDI1 is also expressed (Figure 6c). Collectively, these data indicate that G173V RhoGDI1 can compete with endogenous RhoGDI1 for the interaction with ephrinB1, and this may abrogate the downstream signaling elicited by the stimulation of ephrinB1 with EphB2-Fc.

Therefore, we next examined whether the expression of G173V RhoGDI1 affects EphB2-Fc-mediated RhoA activation. Indeed, the treatment with EphB2-Fc caused RhoA activation in ephrinB1-expressing cells, while this activation was markedly suppressed when G173V RhoGDI1 was also expressed (Figure 6d). To test the effect of G173V RhoGDI1 on EphB2-Fc-mediated cell migration and invasion, we transfected HeLa cells stably expressing wt ephrinB1 with a control vector or G173V RhoGDI1 and performed transwell migration or invasion assays. The stimulation of ephrinB1 by EphB2-Fc augmented cell migration and invasion in HeLa cells transfected with the control vector. In contrast, the enhanced cell migration and invasion stimulated by EphB2-Fc was markedly reduced in HeLa cells transfected with G173V mutant RhoGDI1 (Figures 6e and f). Collectively, these data indicate that G173V RhoGDI1 prevents EphB2-Fc-mediated activation of RhoA, as well as cell migration and invasion.

### Depletion of ephrinB1 by siRNA abates EphB2-Fc-induced RhoA activation, cell migration and cell invasion

**Figure 7:**
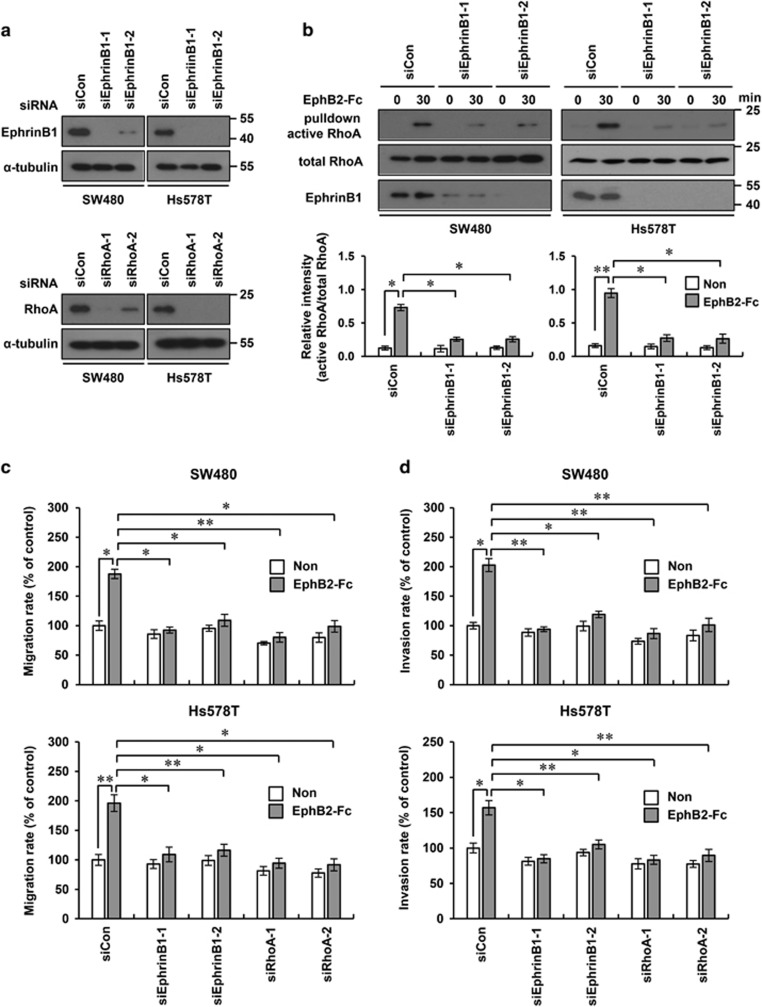
Depletion of ephrinB1 or RhoA by siRNA suppresses cell migration and invasion by EphB2-Fc in human cancer cell lines. (**a**) SW480 or Hs578T cells were transfected with the control siRNA, two ephrinB1 siRNAs or two RhoA siRNAs and analyzed by Western analysis using ephrinB1 and RhoA antibodies. (**b**) Indicated cells were transfected with the control or two ephrinB1 siRNAs. 30 h after transfection, cells were serum-starved for 18 h and then treated with 2.5 μg/ml of EphB2-Fc for 30 min. The pull-down assay using Rhotekin-agarose was performed as described in Materials and Methods. Samples and total lysates were immunoblotted with indicated antibodies (upper panel). Relative intensities were measured by ImageJ and calculated using active RhoA: total RhoA ratios (histograms). Data represent the mean±s.d. of three individual experiments. *P<0.05, **P<0.01. (**c**, **d**) Indicated cells were transfected with the control, two ephrinB1, or two RhoA siRNAs. 36 hrs after transfection, cells were serum-starved overnight and then subjected to migration (**c**) and invasion (**d**) assays as described in Materials and Methods. Data represent the mean±s.d. of three individual experiments. *P<0.05, **P<0.01.

Once we established that in an exogenous expression system EphB2-Fc promotes the activation of RhoA and cell migration as well as cell invasion through an ephrinB1/RhoGDI1 interaction, we next assessed whether depletion of endogenous ephrinB1 or RhoA influences cancer cell migration and invasion. To address this possibility, we transfected the control siRNA, two ephrinB1 siRNAs or two RhoA siRNAs into SW480 or Hs578T cells that express endogenous ephrinB1 and RhoA. Western analysis showed that ephrinB1 siRNAs and RhoA siRNAs significantly reduced the level of ephrinB1 and RhoA protein, respectively (Figure 7a). We tested whether ephrinB1 depletion affects RhoA activation induced by EphB2-Fc. As expected, the stimulation with EphB2-Fc caused RhoA activation in SW480 and Hs578T cells that were transfected with control siRNA, while this activation was markedly suppressed when ephrinB1 was depleted (Figure 7b). Next, we determined whether knockdown of ephrinB1 or RhoA affects EphB2-Fc-induced cell migration and invasion. The stimulation with EphB2-Fc increased migration and invasion in SW480 and Hs578T cells transfected with the control siRNA. In contrast, the EphB2-Fc promoted enhancement of cell migration and invasion was substantially reduced in SW480 and Hs578T cells transfected with two ephrinB1 siRNA or two RhoA siRNA (Figures 7c and d). These results provide evidence that the stimulation of ephrinB1 by EphB2-Fc promotes cell motility and invasiveness through RhoA activation in human cancer cell lines.

## Discussion

The Eph/ephrin system is known to regulate cell–cell adhesion and cell migration in a variety of normal biological processes during development, and dysregulation of this system is involved in cancer progression.^43^ Upon cell-to-cell contact, ephrinB transmembrane ligands transmit signaling events through their cognate Eph receptor tyrosine kinases (‘forward’ signaling), but may also transduce ‘reverse’ signals through their own intracellular domain.^3^ Although several studies provide evidence that the RhoGTPase family of proteins have a crucial role in ephrinB reverse signaling that affects cell movement,^2, 13, 18^ the precise mechanism of how ephrinB1 regulates RhoGTPases remains to be thoroughly investigated.

Several properties of malignant progression have been attributed to aberrant signaling through RhoGTPase proteins.^26^ RhoGDIs provide an expanded level of regulation through controlling subcellular localization of RhoGTPases and their interactions with GEFs, GAPs or effector proteins.^23^ The displacement of RhoGDIs from RhoGTPases is an important step allowing inactive RhoGTPases to become associated with the membrane and be activated by RhoGEFs. A number of studies suggest a role for RhoGDIs in spatial and temporal activation of RhoGTPases.^23^ For example, although RhoGDI1 interacts with Rac1 and blocks the binding to GEFs or effectors, release from RhoGDI1 permits Rac1 to bind its effectors within localized domains where integrin is found.^44^ Our study shows that the stimulation of ephrinB1 with EphB2-Fc promotes the interaction with RhoGDI1 and ephrinB1, which disrupts the RhoGDI1/RhoA interaction (Figures 3 and 4). Thus, RhoA activation in response to engagement of the EphB2 receptor with ephrinB1 may be attributable, at least in part, to displacement of RhoGDI. In line with this concept, both the p75 neurotrophin receptor and TROY have been shown to bind RhoGDI.^39, 40^ Moreover, the interaction between RhoGDI and these proteins is enhanced by their ligands, MAG and Nogo, resulting in the release of RhoGDI1 from RhoA, thus activating RhoA.^39, 40^ Interestingly, although ephrinB1 reverse signaling promotes the release of Rac1 as well as RhoA from RhoGDI1 (Figures 4d and 6a), it only enhanced activation of RhoA (Figures 4a and b). These results suggest that the ephrinB1-mediated displacement of RhoGDI1 from RhoGTPases is not sufficient for activation of RhoGTPases, but clearly contributes to activation of specific Rho family GTPases. EphrinB1-mediated activation of RhoGTPases most likely also requires the recruitment of specific Rho guanine nucleotide exchange factors.

This concept is supported by our previous study,^18^ where we demonstrated that ephrinB1 interacts with a scaffold protein, CNK1, which binds to p115RhoGEF, a Rho-specific guanine nucleotide exchange factor.^18^ Moreover, we showed that in cell lines, adhesion to fibronectin promotes Src-mediated phosphorylation of CNK, which in turn interacts with ephrinB1 in the absence of Eph receptor engagement. The interaction between ephrinB1 and CNK1 links p115RhoGEF and RhoA with MKK4 (an ephrinB1-associated protein) that promotes JNK activation as well as cell migration.^18^ However, in our current study we found that depletion of CNK1 also prevented ephrinB1-mediated RhoA activation upon stimulation with EphB2-Fc (Supplementary Figure 1), suggesting that other factors such as CNK1/p115RhoGEF interaction as well as the release of RhoGDI1 may be required for ephrinB1-mediated RhoA activation.

Our current study identifies the RhoGDI1 as a binding partner of ephrinB1, and finds that an 8-aa region in the ephrinB1 intracellular tail (aa 327–334) is necessary for the interaction with RhoGDI1. The treatment with EphB2-Fc enhanced the interaction of ephrinB1 with RhoGDI1 and promotes RhoA activation in HeLa cells expressing wild-type ephrinB1 (Figure 4b). In contrast, expressing the Δ327–334 ephrinB1 mutant that fails to bind RhoGDI1 does not induce RhoA activation by EphB2-Fc (Figure 4b). Furthermore, overexpression of G173V RhoGDI1, a mutant that does not bind Rho family members, attenuates the interaction of ephrinB1 with endogenous RhoGDI1 and suppresses RhoA activation by EphB2-Fc. Knockdown of ephrinB1 in SW480 and Hs578T human cancer cell lines suppresses RhoA activation by EphB2-Fc (Figure 7b). Therefore, EphB2-Fc-induced activation of RhoA via ephrinB1 may be ascribed to the association of ephrinB1 with RhoGDI1. Interestingly, RhoGDI1 displays a robust interaction with ephrinB1 (Figure 1), and RhoA activity is enhanced by overexpression of ephrinB1 (Supplementary Figure 2A) even in the absence of EphB2-Fc in 293T cells. We discovered that the EphB2 receptor was expressed in 293T cells but not HeLa cells (Supplementary Figure 2B), suggesting that the endogenous EphB2 receptor may stimulate ephrinB1 reverse signaling in 293T cells. Interestingly, ephrinB1 engagement with the extracellular domain of its cognate EphB2 receptor is known to lead to tyrosine phosphorylation of the ephrinB1 cytoplasmic domain, which often leads to recruitment of signaling molecules affecting cell adhesion and movement.^3^ Since, the ephrinB1 mutant with all the intracellular tyrosines replaced by phenylalanine still binds RhoGDI1 with similar avidity (Figure 3b), and the association between CNK1 and ephrinB1 is dependent upon CNK1 tyrosine phosphorylation but not ephrinB1 phosphorylation,^18^ ephrinB1-induced RhoA activation is likely independent of tyrosine phosphorylation.

Ephrin ligands and their Eph receptors have important roles in developmental processes and homeostasis. They are also key modulators of the cancer microenvironment through various mechanisms and are considered as therapeutically targeted for anticancer treatment. EphrinB1 reverse signaling is implicated in regulating cell migration, cell invasion, angiogenesis, metastatic progression of cancer cells, and chemoresistance.^5, 45^ The intracellular domain of ephrinB1 contains docking sites for various signaling components that have a pivotal role in cell migration. Here, we show that the stimulation of ephrinB1 by EphB2-Fc promotes migration and invasion of cancer cells through RhoA activity. Knockdown of ephrinB1 in SW480 and Hs578T cells suppressed cell migration and invasion as well as RhoA activation by EphB2-Fc (Figure 7). Moreover, the Rho kinase inhibitor (Figures 5c and d) as well as knockdown of RhoA (Figures 7c and d) attenuated ephrinB1-mediated cell migration and invasion. We provide strong evidence that an association between ephrinB1 and RhoGDI1 is responsible for EphB2-Fc-induced cell migration and invasion. EphB2-Fc promoted cell migration and invasion in HeLa cells that express wild-type ephrinB1, but not in HeLa cells expressing the Δ327–334 mutant of ephrinB1 that fails to interact with RhoGDI1 (Figures 5a and b). Furthermore, overexpression of a G173V mutant of RhoGDI1 (that can still interact with ephrinB1, but does not interact with the RhoGTPase family of proteins) suppressed cell migration and invasion by wild-type ephrinB1-bearing HeLa cells (Figures 6e and f). Interestingly, a synthetic peptide corresponding to amino acids 331-346 derived from ephrinB1 suppresses RhoA activation and gastric cancer cell migration and dissemination.^46^ This peptide represents the C terminus, including four amino acids that overlap with the critical region identified here (aa 327–334) that is required for the interaction with RhoGDI1 (46; Figure 2c). This overlap suggests the possibility that the synthetic peptide may suppress RhoA activation through the interaction with RhoGDI1, as well as the potential inhibition of an interaction with dishevelled.

Collective and single cell migration depends upon coordination of cell adhesion and motility that requires precise spatiotemporal regulation of the RhoGTPase family members. Cancer cells generally use two modes of migration; one is mesenchymal migration where a partial loss of cell polarity occurs along with the development of a fibroblast-like morphology, and another is ameboid migration in which polarity is completely disrupted and protrusions form and align in the direction of migration.^47^ Eph/ephrin interactions can affect contact inhibition of locomotion (CIL) where migrating cells pause in response to contact with other cells, which leads to retraction of cell protrusions, repolarisation, and cells migrate away from each other. RhoA activation is an important regulator in this event, and thus has an important role in Eph/ephrin control of cancer cell migration. For example, during heterotypic collisions between PC3 prostate cancer cells and fibroblasts, initiation of EphB/ephrinB signaling can suppress CIL and block the repulsive cues elicited by EphA/ephrinA signals.^48^ This activity allows PC3 migration across fibroblasts promoted by the establishment of filopodia and lamellipodia.^48^ In other circumstances Eph/ephrin signaling has been shown to switch the invasive mesenchymal phenotype to an ameboid phenotype via RhoA activation.^49^ Although the signaling from the Eph receptors, rather than ligands, have been studied in most cases, we believe our current study suggests a possible role that ephrinB reverse signaling may play in migration and invasion. Upon contact with the Eph receptor bearing cell, ephrinB1 expressed at the surface of the cancer cell may enhance RhoA activation by engaging RhoGDI, thus removing this negative regulator from RhoA, and leading to migration and invasion of the host cancer cells. Several studies have indicated that the expression of ephrinB1 is upregulated in multiple cancers, such as osteosarcomas, hepatocellular carcinoma, gastric adenocarcinoma, and medulloblastoma.^50, 51, 52, 53^ The present study has revealed an important mechanism by which ephrinB1 regulates cancer cell aggressiveness through Rho activity. Our results pose the possibility that specific inhibition of the ephrinB1/RhoGDI1 interaction may provide a potential therapeutic approach to the treatment of cancer.

## Materials and methods

### Plasmids and reagents

Human RhoGDI1 and RhoGDI2 cDNA was purchased from Origene (GenBank accession: NM_004309 and NM_001175). Flag-tagged RhoGDI1 and RhoGDI2 were amplified by PCR and PCR products were cloned into Pcdna3.1. HA-tagged wt ephrinB1 and various mutants of ephrinB1 (wt, Δ4, Δ20, Δ30, Δ34, Δ60 and TmCyt) have been described previously.^18^ Other deletion mutants of ephrinB1 (Δ287–326, Δ327–334, Δ335–342) and substitution mutant of RhoGDI1 (G173V) were generated by site-directed mutagenesis using the QuickChange methodology (Agilent Technologies, Santa Clara, CA, USA, 200524). Rho kinase inhibitor was obtained from Calbiochem (555550) (Billerica, MA, USA).

### Cell lines, culture and production of stable transfectant cell lines

HEK293T and HeLa cells were a gift from Dr Deborah K Morrison (NCI-Frederick, NIH, Frederick, MD, USA) and were validated by STR profiling (NCI-Frederick). The laboratory of Dr Brad St Croix (NCI-Frederick, NIH) provided MDA-MB-436, DLD1 and LS174T originally purchased from American Type Tissue Culture Collection (ATCC, Manassas, VA, USA). HCT15 and HCT116 cells were provided by Dr St. Croix, and were originally obtained from the NCI repository (NCI-Frederick, NIH). HT-29 cells were obtained from NCR Repository (NCI-Frederick, NIH). MCF-7, MDA-MB-231, MDA-MB-468, Hs578T, SKBR3, T47D, SW480 cells were obtained from the ATCC. SW480 and Hs578T cell lines were validated by STR profiling (NCI-Frederick, NIH). Cells were maintained in DMEM supplemented with 10% fetal bovine serum and antibiotics at 37 °C in a humidified, 5% CO_2_/air atmosphere. 2.5 μg/ml of EphB2-Fc or control Fc (R&D systems, Minneapolis, MN, USA) was pre-clustered with human IgG, as described previously.^16^ Cells were subjected to serum starvation overnight and Eph-Fc introduced into the culture medium at a concentration of 2.5 μg/ml for the indicated times. Stable HeLa cell lines were produced using lentivirus as previously described.^18^ In brief, HEK293T cells were transfected with pCDH-CMV-EF1-puro vector alone or containing wild-type ephrinB1, or the Δ327–334 mutant of ephrinB1, and the culture medium containing lentivirus was harvested after 48 h and used to infect HeLa cells with the addition of 8 μg/ml of polybrene. To generate stable cell lines, infected cells were selected with 2 μg/ml of puromycin (Clontech, Mountain View, CA, USA) for 2 weeks.

### Immunoprecipitation and western blot analysis

Cells were harvested in lysis buffer (20 mm Tris-HCl pH 8, 137 mm NaCl, 2 mm EDTA and 1% NP-40) with protease inhibitor cocktail at 4 °C. Equal amounts of protein lysates were incubated with control IgG, anti-HA (ABM, Richmond, BC, Canada, G036), anti-Flag (Sigma, Allentown, PA, USA, F1804), or anti-ephrinB1 (R&D systems, AF473) at 4 °C overnight under mild agitation. Protein A/G-agarose beads (Santa Cruz Biotechnology, Santa Cruz, CA, USA) were then incubated for 1 h, followed by washing three times with lysis buffer. Immunoprecipitates and total protein lysates were separated by SDS–PAGE and immunoblotted with anti-HA-HRP (Roche, Billerica, MA, USA, 12013819001), anti-Flag-HRP (Sigma, A8592), anti-V5 (Invitrogen, Carlsbad, CA, USA, R961-25), anti-ephrinB1 (R&D Systems, af473) or anti-ephrinB (Santa Cruz Biotechnology C18, SC-910), anti-EphB2 (R&D Systems, AF467), anti-RhoGDI1 (Santa Cruz Biotechnology, SC-360) and anti-RhoA (Santa Cruz Biotechnology, SC-418), anti-Rac1 (Millipore, Billerica, MA, USA, 05-389), anti-Cdc42 (BD Bioscience, San Jose, CA, USA, 610929) and α-tubulin (Sigma, T6199).

### RNA interference

siRNA-mediated depletion was accomplished using AccuTarget Negative Control siRNA (siCon), two different ephrinB1 siRNAs (1045997: siEphrinB1-1, 1046000: siEphrinB1-2) and two different RhoA siRNAs (1129127V: siRhoA-1, 1129130V: siRhoA-2) that were purchased from Bioneer. Transient transfection of siRNAs was achieved using Lipfectamin RNAiMAX (Invitrogen) followed by manufacturers’s instructions. After incubation for 2 days, cells were lysed and efficiency of siRNA was confirmed by immunoblotting using ephrinB1 and RhoA antibodies.

### RhoGTPases activity assay

RhoGTPases activity was assessed using a Rho assay reagent (Millipore, 14-383) and Rac/Cdc42 assay reagent (Millipore, 14-325) as indicated by the manufacturers protocol. Briefly, serum-starved cells were stimulated with EphB2-Fc and then lysed using MLB lysis buffer in the presence of protease inhibitors for 15 min. Rhotekin-agarose or PAK1-agarose was added to cell lysates at a 1:1 volume ratio and incubated at 4 °C for 1 h. After brief centrifugation, the bead pellets were washed with MLB buffer. Laemmli sample buffer was added to the samples and separated by 15% SDS–PAGE, and immunoblotted with anti-RhoA, anti-Rac1 or anti-Cdc42 antibody.

### Cell migration and invasion assays

Transwell cell migration assays were performed using BD Falcon Cell Culture Inserts. Cells were pre-incubated in serum-free medium overnight. 1 × 10^5^ cells were placed in the insert and allowed to migrate for 18 h. The outer chamber was filled with 600 μl of medium containing 10% FBS. After incubation, non-migrating cells on the upper surface of the insert were removed with cotton swab. Migrated cells were fixed and stained with crystal violet, and then eluted with 10% acetic acid. Absorbance was measured at 595 nm using a microplate reader.

In the invasion assay, 2 × 10^5^ cells were seeded in the Matrigel-coated inserts and allowed to invade for 36 h. Invasive cells were stained and analyzed as described above.

### Statistical analysis

Data were obtained from at least three independent experiments performed in triplicate and analyzed using Student’s *t*-test. Results were considered significant when *P*-values were <0.05.
