# Supplementary figures and images for: EphrinB1 promotes cancer cell migration and invasion through the interaction with RhoGDI1
Source: Oncogene. 2017 Oct 23;37(7):861–72. doi: 10.1038/onc.2017.386 (PMC5814325; doi:10.1038/onc.2017.386)

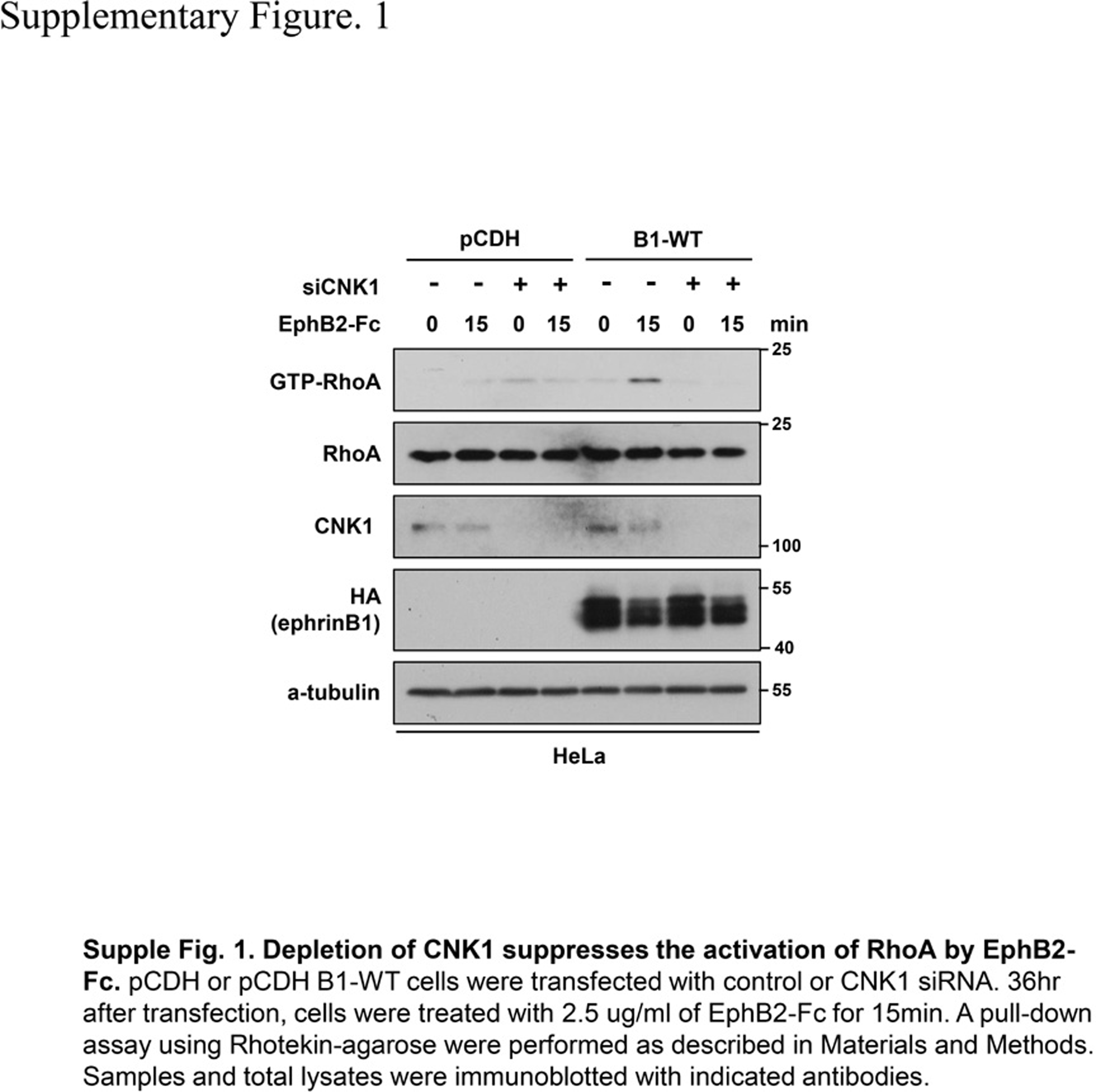

Supplement: Supplementary Figure 1 [file onc2017386x1.tif]

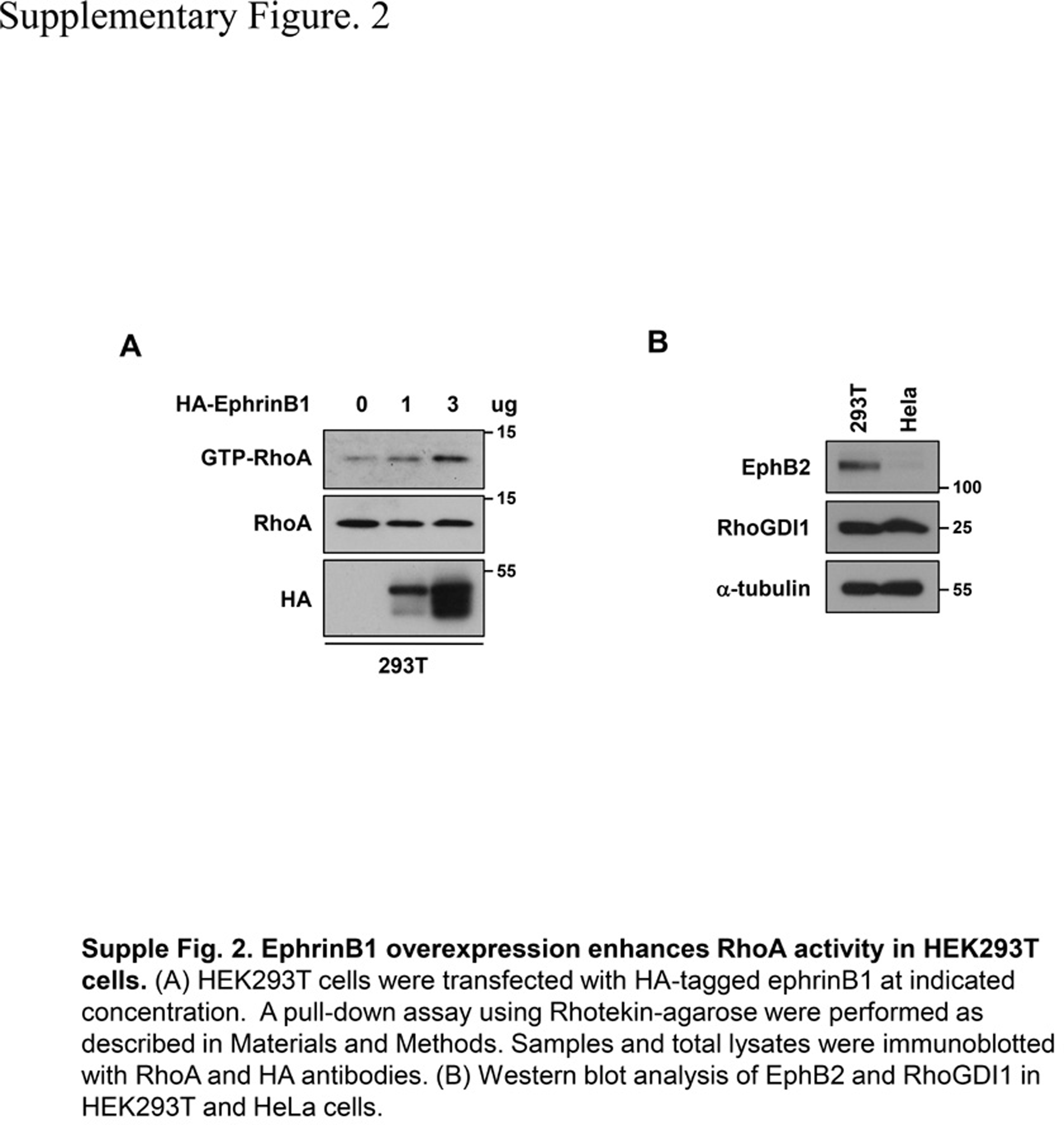

Supplement: Supplementary Figure 2 [file onc2017386x2.tif]
